# Educational field, economic uncertainty, and fertility decline in Finland in 2010–2019

**DOI:** 10.1093/esr/jcae001

**Published:** 2024-01-31

**Authors:** Julia Hellstrand, Jessica Nisén, Mikko Myrskylä

**Affiliations:** Helsinki Institute for Demography and Population Health, Helsinki, Finland; Max Planck Institute for Demographic Research, Rostock, Germany; Helsinki Institute for Demography and Population Health, Helsinki, Finland; Max Planck Institute for Demographic Research, Rostock, Germany; INVEST Research Flagship, University of Turku, Turku, Finland; Helsinki Institute for Demography and Population Health, Helsinki, Finland; Max Planck Institute for Demographic Research, Rostock, Germany; Max Planck—University of Helsinki Center for Social Inequalities in Population Health, Rostock, Germany and Helsinki, Finland

## Abstract

Fertility declined sharply and unexpectedly in Finland in the 2010s across educational levels. Using Finnish register data, we calculated total fertility rates (TFRs) and the proportion of women expected to have a first birth in 2010–2019 for 153 educational groups—reflecting field and level—and estimated how the characteristics of a group predicted its decline. As the educational field predicts factors related to economic uncertainty, heterogeneity in fertility decline across fields could shed light on the role of economic uncertainty behind the recent fertility decline. In general, women with the highest initial fertility levels (health, welfare, and education) and women in agriculture experienced weaker fertility declines (around −20% or less), while women with the lowest initial levels (ICT, arts and humanities) experienced stronger declines (around −40% or more). The extent of the fertility decline increased with higher unemployment and lower income levels in the field and with a lower share employed in the public sector. These uncertainty measures together explained one-fourth of the decline in TFR and two-fifths of the decline in first births. The results imply that fertility declined across all groups, but those with stable job prospects escaped very strong declines. Objective economic uncertainty is one aspect that mattered for the recent fertility decline.

## Introduction

Finland and the Nordic countries have a long history of relatively high fertility (Andersson *et al.*, 2009). However, while female fertility differences across levels of education are small in Finland (in other Nordic countries, the gradient disappeared by the cohorts born in the 1970s (Jalovaara *et al.*, 2019)), the high average fertility levels mask large variations across the fields of education. In many countries, including the Nordic countries, women in the fields of health and teaching form a class of their own, with the highest levels of fertility and the lowest levels of childlessness (Hoem, Neyer and Andersson, 2006b; Begall and Mills, 2012; Michelmore and Musick, 2014; Oppermann, 2017). This is often attributed to the better working conditions and more supportive work–family environment in these fields (Hoem, Neyer and Andersson, 2006a), but also to the higher family orientation of women in these commonly female-dominated fields (Van Bavel, 2010). In turn, the fields of arts and humanities are characterized by low fertility and high levels of childlessness. Given that gender equality and family–work compatibility are explicit policy goals in the Nordic countries, the differences in fertility by field of education caused by structural factors represent an important policy concern, as they suggest that family–work reconciliation may not be as easy for women in all fields (Rønsen and Skrede, 2010).

During the 2010s, the Nordic countries have witnessed pronounced fertility declines, which cannot be fully explained by fertility postponement (Hellstrand *et al.*, 2021). The decline was particularly strong in Finland, where the total fertility rate (TFR) fell from 1.87 in 2010 to 1.35 in 2019 [Official Statistics of Finland (OSF), 2020a]. The Nordic fertility decline appears to be a relatively universal phenomenon; beyond the much faster reduction in first births among childless women than in subsequent childbearing among mothers (Hellstrand *et al.*, 2021), previous studies have not found large variation across other population subgroups (Campisi *et al.*, 2020; Ohlsson Wijk and Andersson, 2022). However, the first birth decline has been somewhat more pronounced among the lower educated (Comolli *et al.*, 2021; Hellstrand, Nisén and Myrskylä, 2022) and those with a weaker labour market attachment (Ohlsson Wijk and Andersson, 2022). Currently, the drivers of the decline remain poorly understood. No studies have examined how fertility in the 2010s has declined by field of education. Besides the importance of monitoring fertility variation across fields in times of changing overall fertility levels, differences in the extent of the decline by field could provide clues of the mechanisms underlying the decline.

Cross-sectional Finnish surveys suggest that perceived uncertainty together with preferences for a child-free life were important reasons for postponing childbearing in the 2010s (Savelieva, Jokela and Rotkirch, 2023). The field of education strongly predicts factors related to economic uncertainty, such as future employment conditions, income security, and work environment (Kogan and Müller, 2003; Salas-Velasco, 2007; Begall and Mills, 2012). It is possible that more stable employment conditions within a field have hindered strong declines. In such a case, we might observe slower fertility declines in the fields such as health and teaching, which are fields with both high employment rates and high shares employed in the public sector. The somewhat stronger decline observed in previous studies among the least educated and those with a weaker labour market position suggest that objective economic uncertainty may matter for the declines, but more evidence is needed.

However, rather similar declines across fields are also possible. The Narrative Framework developed in response to the fertility changes in Europe in the 2010s hypothesized that uncertainty stemming from expectations and perceptions of the future rather than one’s own current circumstances is important in shaping fertility decisions (Vignoli *et al.*, 2020a). The background of this theory is that, across European countries, the strength of the fertility decline in the 2010s was not related to standard factors explaining fertility change (e.g., severity of recession or extent of family policies). Fertility declined with varying intensity in Western and Northern European countries with relatively high fertility levels in the early 2010s and even increased in some Central and Eastern European countries with relatively low fertility levels (see Rindfuss, Choe and Brauner-Otto, 2016). A similar logic could be applied to different fields of education in a country—the extent of the declines might not necessarily be related to objective uncertainty within fields but more so to broader perceived uncertainty irrespective of the actual circumstances.

This study examined how fertility declined by field of education in Finland since 2010. We aimed first to study (i) whether the extent of the fertility decline varied across different educational fields, and second (ii) whether the characteristics of a field were related to the extent of its decline, consequently revealing some of the potential mechanisms behind the decline. We hypothesized finding notable variation by field of education, and stronger declines in fields characterized by higher (objective) uncertainty, e.g., lower employment. To test these hypotheses, we used detailed Finnish full population register data and calculated TFRs and expected shares having a first birth between 2010 and 2019 for 153 groups of education distributed over four education levels.^1^

A key focus of our study is the role of economic uncertainty in contemporary fertility dynamics, and the lessons learned bear importance beyond the country under investigation. In this study, we also bridge across literature on fertility differentials by the horizontal dimension of education on one hand and recent literature on economic uncertainty on the other hand. The present aggregate analysis allows the evaluation of fertility patterns and trends for an exceptionally large number of detailed educational fields identified from individual-level data, which has the potential to reveal patterns hidden by broad categorizations. Moreover, this is the first study to provide fertility estimates by field of education in Finland. Finland is an interesting study setting not only because of the strong fertility decline in the 2010s but also because Finland is considered a Nordic vanguard country in family-demographic changes (Ohlsson‐Wijk, Turunen and Andersson, 2020). Therefore, understanding changes in Finland can help shed new light on the ongoing shifts in fertility behaviour more generally, including concurrently occurring fertility declines in a number of other countries. Additionally, the role of public sector employment for fertility is interesting to study, as Finland’s public sector ranks among the largest internationally.

## Background

### Nordic fertility regime and policy challenges

Finland is often situated within the Nordic fertility regime—a concept that refers to the combination of high and stable fertility, strong support for working mothers, and high female labour force participation (Neyer *et al.*, 2006; Merz and Liefbroer, 2018). Within this regime, the Nordic countries share many similar traits in their family policies and childbearing patterns (Andersson *et al.*, 2009; Jalovaara *et al.*, 2019). Gender equality is an explicit policy goal in the Nordic countries (Esping-Andersen, 1990), and family policies are designed to promote gender equality rather than to promote childbearing (Rønsen, 2004). However, it is generally assumed that the family policies have contributed to a favourable setting for childbearing, as they reduce work–family conflicts among mothers (Brewster and Rindfuss, 2000; Adserà, 2004). The dual earner–dual caregiver model prevalent in the Nordic countries is promoted by relatively generous family policies, e.g., earmarked parts of paid parental leave to both parents and affordable childcare (Ellingsæter and Leira, 2006; Gornick and Meyers, 2009).

The combination of high levels of female labour force participation with relatively high average levels of fertility in the Nordic countries suggests that the policy goal to promote gender equality has been successful. However, the relatively high fertility levels have existed concurrently with high fertility variation between educational fields (Hoem, Neyer and Andersson, 2006b), and strong gender segregation in the labour market (Mandel and Semyonov, 2006; Hook *et al.*, 2022). The gendered occupational structure is rooted in gender-typical choices already in education (Prix and Kilpi-Jakonen, 2022). Furthermore, women educated to work in female-dominated occupations mainly in the public sector (e.g., health and teaching) have much higher fertility than women educated in less gender-segregated professions or in male-dominated fields (Lappegård and Rønsen, 2005; Hoem, Neyer and Andersson, 2006b). This indicates that work–family reconciliation might be more difficult in some fields compared to others.

### Educational field and fertility

In most developed countries, higher educated women tend to have less children than lower educated women. This negative gradient by education is often explained by the higher opportunity costs of motherhood among the more highly educated (Oppenheimer, 1994), by selection due to differences in childbearing preferences, and by increased possibilities of lifestyles other than motherhood being available to the more highly educated (Surkyn and Lesthaeghe, 2004). However, the negative educational gradient in fertility is small in Finland (the difference was −0.16 between tertiary and primary educated of the late 1960s cohort, compared with an average of −0.36 for 15 European countries (Nisén *et al.*, 2021)) and has vanished in other Nordic countries for the early 1970s cohort (Jalovaara *et al.*, 2019), often considered to result from the Nordic family policies supporting the combination of work and family (Brewster and Rindfuss, 2000; Adserà, 2004).

An increasing number of studies pay attention to the field of education, which has turned out to be a strong predictor of fertility in high-income countries (e.g., Martín-García and Baizán, 2006; Begall and Mills, 2012) and even stronger than the mere level of education in Nordic countries (Lappegård and Rønsen, 2005; Hoem, Neyer and Andersson, 2006a, 2006b). For instance, in the 1955–1959 Swedish cohorts, among women educated to the lower tertiary level, midwives had on average 2.39 children, while women educated in arts had 1.65 children (Hoem, Neyer and Andersson, 2006a). In comparison, those with only primary education had 2.1 and those with tertiary education had 1.9 children on average (Jalovaara *et al.*, 2019). Similarly, childlessness levels varied from 10% for primary-school teachers to above 30% in humanities among the tertiary educated, but remained close to 15% across the different levels of education (Hoem, Neyer and Andersson, 2006a). Following the more recent cohorts, childlessness has increased particularly among the least educated (Jalovaara *et al.*, 2019).

There are a number of reasons suggested to explain the variation in fertility across different fields of study, and the literature suggests both causal (field of study affects employment prospects and attitudes, and thereby, fertility) (e.g. Cook and Minnotte, 2008) and selection based on preferences (personality traits and attitudes about family life affect both the choice of field and fertility) (e.g., Van Bavel, 2010) effects. Reverse causality is also possible, as childbearing may influence women’s choice of field (Tesching, 2012). Although this study does not aim to test the relative importance of these different mechanisms, in the following we briefly outline the reasons depicted by the literature to shed light on the mechanisms behind fertility variation across fields, which may help to explain potential variation in the strength of recent fertility decline.

### Educational field and labour market

Different fields of education lead to occupations with different characteristics. First, educational fields differ in how challenging it is to enter the labour market. Those who have studied in fields that do not lead to any particular occupation may face difficulties in becoming established in the labour market and are at high risk of unemployment. Examples of such fields are general education, fine arts and humanities, and general social sciences. These fields have very low fertility levels in both the Nordic countries (Hoem, Neyer and Andersson, 2006b) and elsewhere (Michelmore and Musick, 2014).

Second, employment stability varies between educational fields. Fields that lead to jobs in the public sector may have a positive impact on childbearing because they provide stable employment prospects and a secure income. For instance, health care and teaching work is predominantly carried out in the public sector in the Nordic countries. Generally, employment in the public sector is less subject to fluctuations in the economy than employment in the private sector (e.g., Kopelman and Rosen, 2016), as services provided by the public sector (e.g., health and teaching) need to be organized regardless of the business cycle. Moreover, in many countries, the public sector was the first to introduce parental leave and part-time work to facilitate the compatibility of motherhood and employment (Hoem, Neyer and Andersson, 2006a; Feeney and Stritch, 2019). However, a study from Sweden found that the positive association of employment in the public sector with entry into motherhood was limited mainly to caring and teaching occupations (Ohlsson Wijk, 2015).

Income is another aspect that provides security to the family and alleviates the direct costs of childbearing (Becker, 1993). Higher female income has been shown to promote motherhood, particularly in the Nordic countries (Jalovaara and Miettinen, 2013; Andersson, Kreyenfeld and Mika, 2014). Artists, humanists, and librarians are examples of fields with high childlessness and relatively low income compared with other fields with the same education level, but income alone does not explain the variation in fertility across fields in Sweden (highest income was found in male-dominated fields, which had average levels of fertility) (Hoem, Neyer and Andersson, 2006a). Furthermore, skill depreciation during family-related work absence is another potential factor that could explain fertility differences by field of education, as some fields are evolving quicker (e.g., technological fields) than others (e.g., service sector) (Hoem, Neyer and Andersson, 2006a).

### Preferences and social environment

Preferences and self-selection are considered to be important in shaping both career and childbearing paths (Hakim, 2003). Preferences and interests that influence the choice of a specific study field may also be associated with childbearing desires. Hence, women with a stronger family orientation have been argued to choose certain types of fields that emphasize the care of other individuals, such as fields within health, welfare and education (Hoem, Neyer and Andersson, 2006a). In these fields, the work is oriented towards considering the needs of others, which is also the essence of parenting. The social environment during education and in working life is also assumed to affect family-related attitudes and childbearing. Fields that convey stereotypical female qualities are assumed to foster the preference to have (more) children (Hoem, Neyer and Andersson, 2006a; Van Bavel, 2010). In turn, humanities, fine arts, and social sciences are fields that were the first to question norms related to motherhood and childbearing (Neyer, Hoem and Andersson, 2017).

Female-dominated fields in the public sector tend to have generally higher fertility levels, and also fields in the private sector that are highly dominated by women (e.g., food production, textile industries, and beauty and hairdressing) have low levels of childlessness (Neyer, Hoem and Andersson, 2017). Female-dominated workplaces are considered more flexible and considerate of needs to care for children. In turn, women educated in fields with a more balanced distribution of men and women (e.g., business and journalism) are assumed to face more competition from men at the workplace, which is more likely to discourage childbearing (Neyer, Hoem and Andersson, 2017). Fields with higher competition and/or a steeper earning profile could be assumed to lead to higher motherhood penalties of long-care leaves (Van Bavel, 2010). Furthermore, women who choose the most gender-atypical fields may be less likely to conform to traditional gender roles and childbearing patterns (Ohlsson Wijk, 2015). Indeed, women across several European countries with more traditional gender role attitudes were less likely to postpone their first birth in the early 2000s (Van Bavel, 2010). Generally, it could be assumed that a higher share of females in a field reflects easier work–family compatibility and a less competitive working environment, but also higher childbearing preferences.

### Economic uncertainty and fertility decline

Economic uncertainty is an important factor in explaining trends in fertility patterns (e.g., Kreyenfeld, 2010; Kreyenfeld and Andersson, 2014). Empirical evidence shows that fertility trends tend to follow business cycles; individuals often postpone childbearing in economically uncertain times and favour it in times of economic growth (Sobotka, Skirbekk and Philipov, 2011). Of all parties, particularly the first births tend to be affected by economic uncertainty (Blossfeld and Hofmeister, 2006; Kreyenfeld and Andersson, 2014). In line with this earlier evidence, the fertility declines in the 2010s began in the aftermath of the Great Recession. However, the declines continued after macro-economic recovery.

Perceived uncertainty of the future has then been introduced as a framework to explain fertility patterns (Comolli *et al.*, 2021). Scholars argue that uncertainty, which is not necessarily rooted in objective dimensions of economic uncertainty, has increased in the 2010s due to globalization dynamics, new technologies, media channels, migration flows and ever-growing concerns about climate change (Vignoli *et al.*, 2020b; Neyer *et al.*, 2022). Individuals shape expectations and perceptions of the future based on their past experiences and the social context (e.g., shared narratives from peers and the media) in which they live—and not necessarily on their own current economic situation. This future outlook is suggested to play an increasingly important role in shaping fertility decisions (Vignoli *et al.*, 2020a).

This idea of perceived uncertainty is supported by empirical evidence from the Nordic countries showing pronounced fertility declines also in high education and income groups (Comolli *et al.*, 2021; Hellstrand, Nisén and Myrskylä, 2022), groups that tend to experience less objective economic uncertainty. Additionally, Finnish surveys find that perceived uncertainty was one of the main self-reported reasons for postponing childbearing in the 2010s (Savelieva, Jokela and Rotkirch, 2023). However, the first birth decline accelerated more in the less highly educated groups (Comolli *et al.*, 2021) and among those with the weakest labour market attachment (Ohlsson Wijk and Andersson, 2022). These findings suggest that, in addition to perceived uncertainty, actual economic constraints also play a role in the recent changes.

We extend this line of research by focusing on the aspect of uncertainty in the study of fertility across educational fields. More precisely, we look into the fertility decline in Finland by field of study with different degrees of objective economic uncertainty, as measured by quantifiable characteristics of the fields. We hypothesize that in times of increasing perceived uncertainty, combined potentially with a child-free life becoming more popular (Golovina *et al.*, 2023), those educated in fields with more uncertain labour market prospects might be more prone to postpone their childbearing. On the other hand, as suggested by the Narrative Framework, in case the perception of uncertainty is not strongly linked to such objective measures, the variation in the fertility decline by field would not be explained by our measures of uncertainty (e.g., share employed, average income, and share working in the public sector).

## The Finnish context

### The education system

The Finnish education system is flexible without early track differentiation, and there are wide possibilities to continue to higher education or to change the field of education (Blossing, Imsen and Moos, 2014). After the 9-year compulsory basic education, students can apply for two different types of upper secondary education: general upper secondary schools or vocational institutions. General upper secondary education prepares for further studies in universities and universities of applied sciences (UAS)^2^ but does not qualify students for any particular occupation. Vocational upper secondary institutions teach basic skills required in the field and often qualify students for particular occupations. It is common to study for multiple vocational degrees; more than half of all new students in vocational education have already completed at least one secondary or higher level of education (Finnish National Agency for Education, 2019).

Education in Finland is free of charge and interruptions to educational careers are not unusual (Orr, Gwosc and Netz, 2011). Furthermore, graduates from vocational upper secondary institutions are eligible to apply for further studies at UAS and may formally qualify for some fields of university education. There are also possibilities to transition between UAS and universities, e.g., to conduct a university Master’s degree after an UAS Bachelor’s degree. The UAS Bachelor’s degrees tend to lead to an occupation, whereas a university Bachelor’s degree rather tends to prepare for Master’s studies (Kilpi, 2011; Blossing, Imsen and Moos, 2014). Notably, the fields of education are heavily gender-segregated in Finland; women constitute almost 90% of the population in the field of health and welfare and almost 80% in the field of education, but at most 20% in the fields of engineering, manufacturing and construction, and ICT (Official Statistics of Finland (OSF), 2020b).

### Unemployment, income levels, and public sector work

The female unemployment rate was 6.2% in 2019 in Finland, right below the EU-27 average of 7.2% (Eurostat, 2022c). During the 2010–2019 period, female unemployment rose from 7.7% to 8.6% in 2015 but dropped below the initial level towards the end of the period. The unemployment rate was 15.9% among women aged 15–24 years and 4.9% among women aged 25–54 years, being 3.6 and 0.4 percentage points, respectively, below the unemployment rate among Finnish men.

Income levels have risen faster among the higher income groups in the 2010s, but income inequalities in Finland remain comparatively small (OECD, 2020). However, gender difference in earnings is high: the median wage for full-time employment was 18% lower for women than for men in 2019, compared with an OECD average of 14% and around 5–7% in other Nordic countries (OECD, 2020).

Finland’s public sector together with that of other Nordic countries ranks the highest of all European countries; public employment constitutes 25% of total employment, compared with 16% in the EU-27, with limited changes over time (Eurostat, 2022b). Just above 40% of all women work in the public sector, and this share has been stable in the last decade (Official Statistics of Finland (OSF), 2022). Of the total public sector, women constitute the majority of workers. Further, the largest groups working in the public sector include health care, education, and social service (e.g., children’s day care, care for the elderly, and social work) (Ministry of Finance, 2006).

## Data and methods

### Categorization of fields

We used register data from Statistics Finland for women born in Finland and aged 15–49 years in 2000–2019 and permanently living in Finland at the end of the year. These individual-level data were used to identify exceptionally detailed groups of educational fields each year during the period of analysis, and for these groups, age-standardized fertility indicators were calculated and further used in an aggregate-level regression analysis. We first used the ISCED 2011 classification to separate between broad field and level of education. We considered four levels of education: primary (ISCED 0–2), secondary (ISCED 3–4), lower tertiary (ISCED 5–6), and higher tertiary (ISCED 7–8). In order to form more detailed groups beyond the ISCED 2011 classification, we used a six-digit code provided by Statistics Finland. Hence, we are able to distinguish, for instance, between nurses, health care providers, and midwives within the broad field of nursing and midwifery (ISCED 0913) and between general teachers and special teachers within the broad field of teacher training without subject specification (ISCED 0113). We consider the highest obtained degree. If an individual has obtained several degrees at similar levels, we consider the most recent one.

We identified in total 153 groups of education, which are shown in Supplementary Appendix Table 1: 45 groups at the secondary level, 55 groups at the lower tertiary level, 52 groups at the higher tertiary level, and one group consisting of those with only primary education. Women educated in the broad field of health and teaching constitute one-third of all women and around 20% are educated in the field of business and social sciences. The fields of engineering, agriculture, ICT, and natural sciences combined constitute almost 13% and the field of services another 13%, followed by general education and broad programmes (12%), and arts and humanities (10%). Of the detailed groups, the largest groups are nursing and business at secondary and lower tertiary levels, social work at tertiary level, and hotel and restaurant at secondary level. At higher tertiary level, the largest groups are women educated in business and as general teachers, physicians, and lawyers.

### Fertility outcome

For the 153 groups of education, we calculated TFRs using 5-year age-specific fertility rates, and the share expected to ever have a first birth (TFRp1) using 5-year age-specific first birth rates (first births per number of childless women) and a lifetable approach. Age is defined at the end of the year and rates are based on the transitions from the end of a year to the end of the following year. In order to increase the stability of rates, we grouped together observations in 2009–2011 (the fertility peak) and 2017–2019 (latest available years). The change in TFR and TFRp1 between 2009–2011 and 2017–2019 is the main outcome of interest for our analysis.

### Independent variables in regression models

Characteristics of the field analysed in this study are the proportion unemployed (i.e., percentage of the labour force without a job), mean annual income^3^ among the employed (on log scale), and the share working in the public sector (of those employed).^4^ These characteristics were measured in 2018^5^ at age 25–29 years to capture the uncertainty level early in the career and at or before the prime childbearing age. The characteristics are measured at age 25–29 years also for the secondary educated, as it is relatively common to earn multiple vocational degrees and consequently, to earn the most recent degree at a relatively high age. However, we perform sensitivity analyses calculating the characteristics at age 20–24 for the secondary educated whenever possible.

### Methods

We use scatter plots and weighted trend lines to illustrate the fertility decline by field and level of education. The weights are based on the size of the educational fields at age 30–34 years. Furthermore, we use weighted multivariate linear regression to analyse the association between the characteristics measuring uncertainty (unemployment, income, and public sector work) and the fertility decline across fields. To compare the predictive power of different measures, the models are fit to normalized data such that each variable has a standard deviation of 1. Finally, we use counterfactual predictions to estimate how much the fertility decline could have been reduced had factors reflecting uncertainty (e.g., unemployment) been low. Women with primary education only are not included in the regression analyses or counterfactual predictions because their education does not qualify them for a particular field. They represent 17% of all women of fertile ages in the study period and their fertility declined by 28% in the period (Supplementary Appendix Table 1).

## Results

### Fertility decline by broad field of education

**Figure 1:**
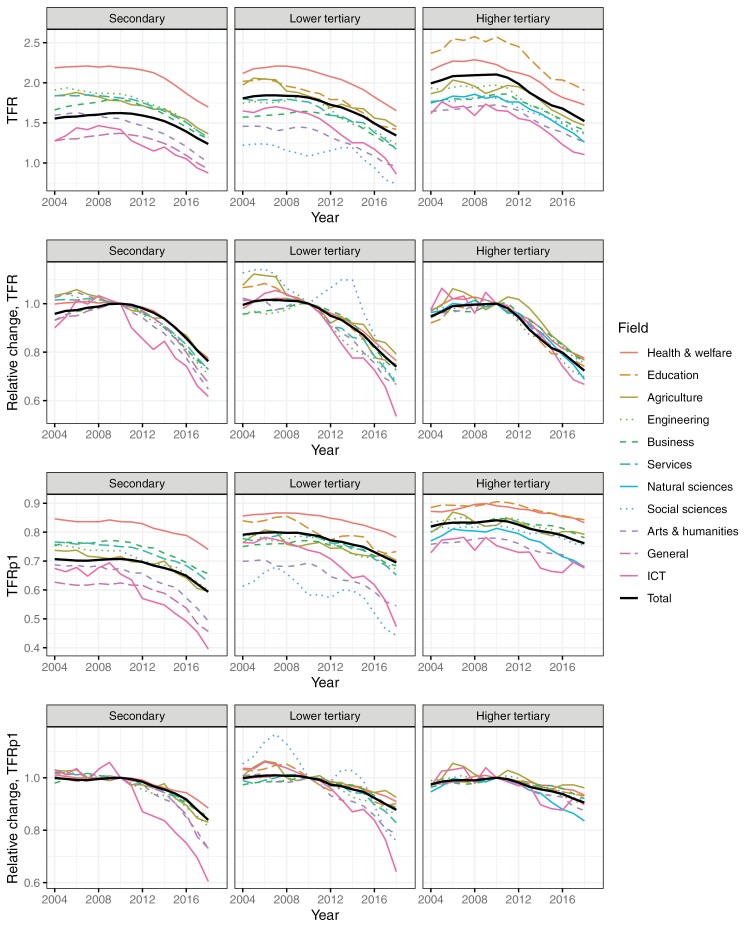
TFR (3-year moving average), relative change (baseline 2010) in TFR, TFRp1 (3-year moving average), and relative change in TFRp1 (baseline 2010) by level and broad field of education in 2004–2019

Figure 1 shows the TFR and the TFRp1 (displayed by 3-year moving averages) in 2004–2019 and the changes relative to 2010 by level and broad field of education. As expected, we observe the highest TFR and TFRp1 levels in health and teaching and the lowest levels in arts and humanities, ICT, and general education at the secondary level. For instance, comparing the levels before the onset of the fertility decline in 2010, among the secondary educated, women in health and welfare had TFR of 2.19 and TFRp1 of 0.84. Correspondingly, women with only general education or those educated in ICT had TFR 1.37–1.42 and TFRp1 0.62–0.65. Among the higher tertiary educated, the TFR and TFRp1 of women educated in teaching were 2.57 and 0.91, respectively, while women educated in ICT and arts and humanities had TFR 1.66–1.72 and TFRp1 0.75–0.78.

Comparing the declines in the 2010s by the level of education, the declines in TFR were typically rather similar, but the declines in TFRp1 were somewhat weaker among the higher tertiary educated. However, the variation in the strength of the decline by field of education was much more pronounced than by level of education; the fields with the lowest levels at the onset of the fertility decline typically had the strongest declines, and the variation across fields appeared larger among the secondary and the lower tertiary educated than among the higher tertiary educated. Women educated in health and welfare and agriculture typically experienced the weakest declines in TFR, around a 21–24% decline at all levels. The strongest declines in TFR were observed in ICT, arts and humanities, and general education at secondary level (33–38%), in ICT at lower tertiary level (more than 40%), and in ICT, natural sciences, and engineering at higher tertiary education (30–33%).

The strength of the decline in TFRp1 varied from 12% in health and welfare, to 27% in general education and arts and humanities, and further to 40% in ICT among the secondary educated. Among the higher tertiary educated, the strength of the decline in TFRp1 varied from 4 to 7% in agriculture, health and welfare, and education; to 10 to 12% in engineering, ICT, and arts and humanities; and to 16% in natural sciences.

In absolute terms, we also observe divergence in the TFRp1 patterns across fields, but for the TFR, the absolute declines are more similar; for instance, among the secondary educated, the absolute change ranges from −0.41 children in agriculture to −0.55 in arts and humanities, and the change in health and welfare amounts to −0.49 children.

### Fertility decline by detailed field of education

**Figure 2:**
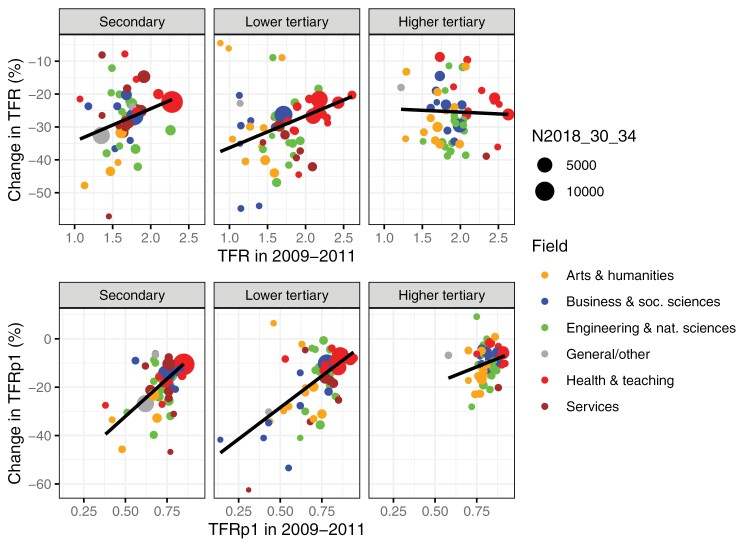
(Top) TFR in 2009–2011 on the x-axis and relative change in TFR in the 2010s on the y-axis by level and detailed field of education. (Bottom) TFRp1 in 2009–2011 on the x-axis and relative change in TFRp1 in the 2010s on the y-axis by level and detailed field of education. The regression slope is weighted by the size of the field. Fields that are related to each other and/or have similar fertility levels are depicted with the same colour for easier reading *Note*: Some of the groups had low numbers of childless individuals in certain age groups, and the number of age groups on which TFRp1 is based therefore differs across fields. Consequently, the very low levels (e.g., 0.14 in law at lower tertiary level in 2009–2011) in some small fields should be interpreted with caution. However, for each educational group, TFRp1 is calculated based on the same number of age groups over time. Furthermore, excluding the small fields where TFRp1 is based on only a few age groups did not significantly change the results.

The relationship between the initial fertility level in 2009–2011 and the relative fertility decline in the 2010s by detailed field of education is illustrated in Figure 2. The top panels show the TFR, and the bottom panels the TFRp1. The exact values can be found in Supplementary Appendix Table 1. In terms of TFR, the strongest fertility declines are in fields with the lowest initial fertility (e.g., fine arts, library science, and ICT) among women educated to the secondary or lower tertiary level. Among women educated to the higher tertiary level, there is no relationship between the initial level and the strength of the decline. In terms of first births (TFRp1), the decline is stronger in fields with lower initial levels regardless of educational level, but this relationship between initial level and decline is much weaker at the higher tertiary level. Like in the case of broad fields, in absolute terms, the TFR declines are rather similar regardless of the initial level, but for the TFRp1, the absolute decline is similar to the relative decline (Supplementary Appendix Figure 1).

### Fertility decline and economic uncertainty in the field

**Figure 3:**
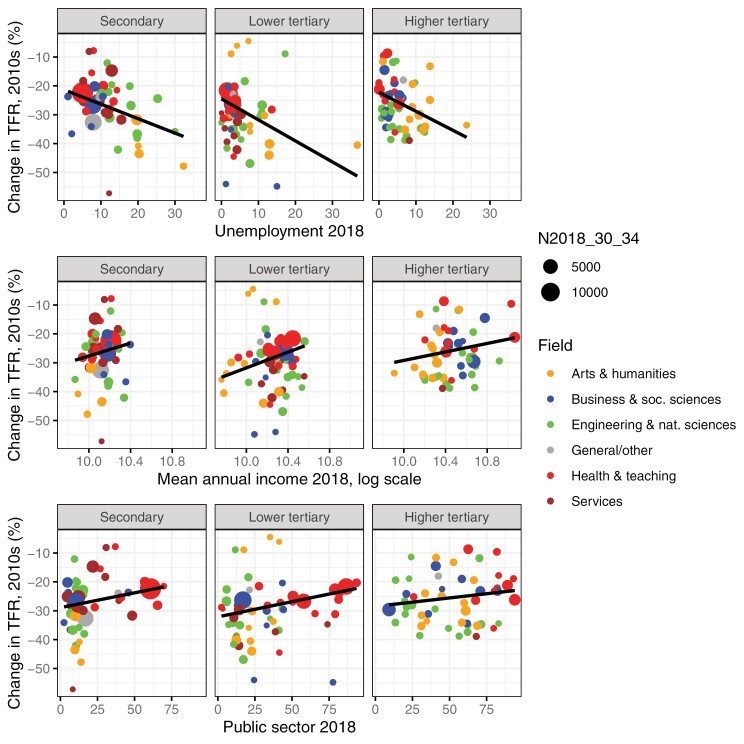
Uncertainty measures and relative decline in TFR in the 2010s

The relationship between the characteristics reflecting economic uncertainty (share unemployed, mean annual income, and share in the public sector) in the field in 2018 and the relative change in TFR and TFRp1 is shown in Table 1 and answers the question of how current characteristics of the fields reflecting uncertainty are associated with fertility declines of a field. The variables are standardized, so that a change of 1 SD in the explanatory variable is associated with a change of β standard deviations of the change in fertility. Scatter plots of these associations based on unstandardized variables can be seen in Figure 3 (TFR) and Supplementary Appendix Figure 2 (TFRp1). In bivariate analyses, the changes in the TFR and in the TFRp1 are associated with all three measures of uncertainty. The strength of the fertility decline increased with higher unemployment, lower mean income, and lower share working in the public sector within the field.

**Table 1:** Regression models estimating the relative change in TFR and TFRp1 in the 2010s. The models are weighted by the size of the field. Number of fields: 152

	Change in TFR (%)				Change in TFRp1 (%)			
	Separate models^a^		Multivariate model^b^^,^^c^	Multivariate model, interactions	Separate models^a^		Multivariate model^b^^,^^c^	Multivariate model, interactions
	Estimate	*R* ^2^	Estimate	Estimate	Estimate	*R* ^2^	Estimate	Estimate
Intercept			0.36***	0.22			0.17*	0.12
Unemployment	−0.40***	0.17	−0.33***	−0.32*	−0.45***	0.37	−0.36***	−0.41***
Log(Income)	0.23**	0.06	0.00	−0.21	0.32***	0.23	0.07	−0.03
Public sector	0.25***	0.14	0.19***	0.19	0.19***	0.23	0.12**	0.16
Lower tertiary			−0.54***	−0.42*			−0.11	−0.02
Higher tertiary			−0.35	−0.23			0.13	0.31
Lower tertiary: Unemployment				−0.03				0.20
Higher tertiary: Unemployment				−0.26				−0.03
Lower tertiary:log(Income)				0.22				0.34
Higher tertiary:log(Income)				0.13				−0.01
Lower tertiary: Public sector				0.02				−0.02
Higher tertiary: Public sector				−0.01				−0.12
*R* ^2^			0.24	0.25			0.40	0.42
Adjusted *R*^2^			0.22	0.19			0.38	0.37

^a^In the separate models for each variable, educational level is included in the model, but its coefficients are not shown.

^b^Removing educational level from the model would reduce *R*^2^ by 8 percentage points for the change in TFR and by 1 percentage point for the change in TFRp1.

^c^When the primary educated category is included, the unemployment coefficient decreases slightly, and in the TFRp1 multivariate model, the *R*^2^ increases to 0.48 (adjusted *R*^2^ 0.46).

*The significance levels at 0.05.

**The significance levels at 0.01.

***The significance levels at 0.001.

### Multivariate regression models

The multivariate regression models include all three uncertainty indicators simultaneously and show how much the variables combined explain the variation in the fertility decline between fields (Table 1). These models explain 24% and 40% of the variation in the strength of the decline in TFR and TFRp1 between fields. Net of other uncertainty measures, the associations with unemployment remain the strongest, while the associations with income are no longer significant.^6^ Hence, the fields with high unemployment also tend to have low income among those employed but including both measures does not add information to the model (results not shown). In addition, the link to public sector work remains significant in the multivariate models, although the coefficients are somewhat smaller than in the univariate models. We further show the results of the uncertainty model including interactions with education level, and this model is used in the prediction and counterfactual analysis.

Sensitivity analyses show that the results are rather similar regardless of when the characteristics are measured (2010 versus 2018) and that the declines are generally not associated with changes in the characteristics over time (with the exception of a weak association between increases in unemployment and the decline in first births) (Supplementary Appendix Table 2). When we measure labour market characteristics at age 20–24 for the secondary educated (whenever possible), the association with income becomes stronger and significant, whereas the association with unemployment becomes somewhat weaker (Supplementary Appendix Table 3). However, the explanatory power remains relatively similar.

In Supplementary Appendix Tables 4 and 5, we show how the uncertainty model is influenced by the inclusion of other factors strongly associated with the decline of a field, such as proportion of students,^7^ occupational match, the gender composition, and the proportions in unions. The decline is weaker in fields with a larger proportion of students or females and stronger with lower occupational match and lower proportions in unions. The associations between uncertainty measures with the decline remain, for most parts, similar when these other factors are controlled for. However, union status clearly attenuates the associations of uncertainty measures (i.e., including the proportion single in the TFRp1 model halves the link to unemployment, and the association with the public sector is no longer significant). This is not surprising, as the probability of forming a cohabiting union depends on one’s position in the labour market (Jalovaara, 2013). By including the share females, the association with public sector work diminishes and the association with income strengthens. It appears that the gender composition explains the decline rather than public sector employment, but as these two measures are correlated (correlation coefficient between 0.23 and 0.43, respectively, for the secondary and the higher tertiary educated), it is difficult to empirically distinguish their relative importance.

To investigate this further, we stratify the analyses by gender composition (fields with higher versus lower than mean female share) in Supplementary Appendix Table 6. In fields with a lower share of females, the association between unemployment and the strength of the TFR decline appears stronger, and the relationship with public sector work is no longer significant. In the case of TFRp1, the association with unemployment remains strong regardless of the gender composition, but the degree of public sector work seems only important in more female-dominated fields, and income levels somewhat more important in less female-dominated fields.^8^ To sum up, unemployment remains the most important dimension of economic uncertainty associated with the decline, and its association with the decline is robust to the inclusion of other factors.

### Predicted declines and counterfactual scenarios

**Figure 4:**
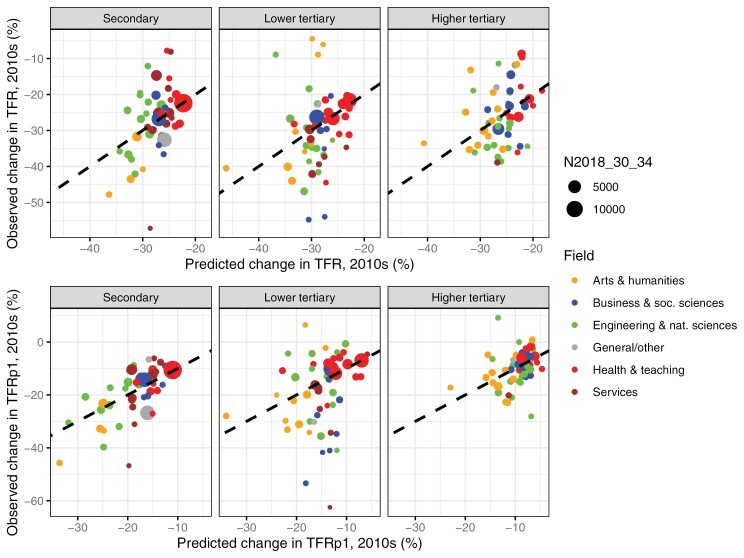
Observed (y-axis) and predicted change (x-axis) in TFR and TFRp1 in the 2010s based on the uncertainty model (with interactions) in Table 1 by level and detailed field of education

The observed and predicted declines based on the uncertainty model (with interactions with educational level) are presented in Figure 4. The uncertainty model shown in Table 1 typically correctly predicted the stronger declines in TFR and TFRp1 in some of the fields in arts and humanities, engineering, and natural sciences (e.g., handicrafts, history, materials, and wildlife), the intermediate decline in business, and the weaker decline in health and teaching. However, the model systematically underpredicted both the most severe declines and the weakest declines in TFR and TFRp1. The model underpredicted especially the strong decline in natural sciences at the tertiary level.

**Figure 5:**
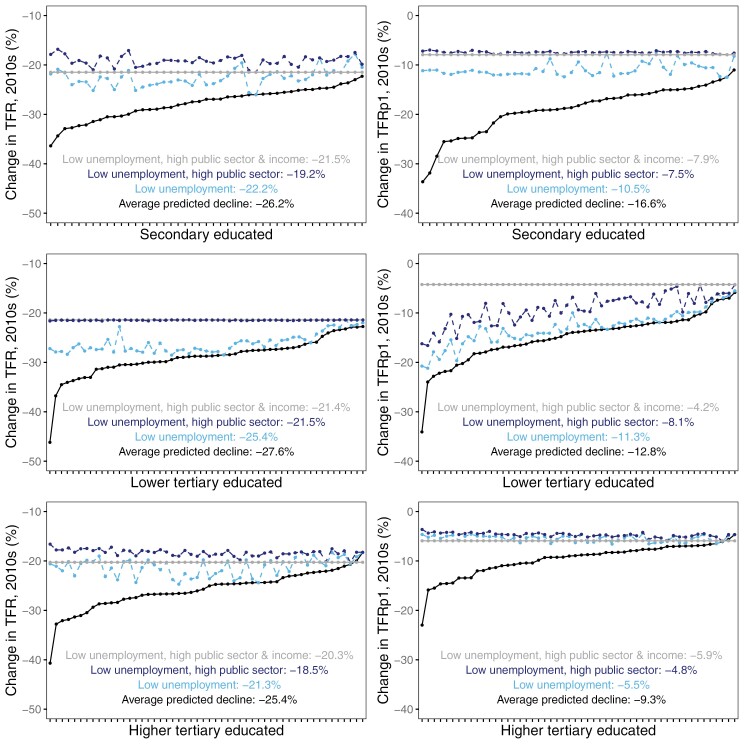
Predicted declines and counterfactual scenarios in change in TFR (left-hand side) and TFRp1 (right-hand side)

We further estimate how much the fertility decline would have been reduced in a counterfactual scenario where uncertainty was low (Figure 5). Unemployment was set to the minimum rate observed at a given level (1.1% at secondary level, 0% at tertiary level), and the share working in the public sector was set to the maximum share observed at a given level (69.7% at secondary level, ~94% at tertiary level). Rather than a plausible future scenario for Finland, this scenario is meaningful in illustrating the differences in the fertility decline associated with these uncertainty factors. The light blue dotted line represents the scenario where unemployment is low, and the dark blue dotted line represents the scenario where both unemployment is low and the share working in the public sector is high. If uncertainty was low in all fields, the decline in TFR would have been reduced on average from −26.2% to −19.2% for the secondary educated, from −27.6% to −21.5% for the lower tertiary educated, and from −25.4% to −18.5% for the higher tertiary educated. Moreover, the decline in TFRp1 would have been reduced on average from −16.6% to −7.5% for the secondary educated, from −12.8% to −8.1% for the lower tertiary educated, and from −9.3% to −4.8% for the higher tertiary educated. Hence, the TFR reduction is mid-sized (a reduction by one-fourth), while the TFRp1 reduction is larger (a reduction by one-half at secondary and higher tertiary levels and one-third at lower tertiary). More than half of this reduction in TFR was due to low unemployment at secondary and higher tertiary levels and up to 67% and 84%, respectively, for the reduction in TFRp1.

### Lifetime fertility and the link to changing field

To compare the period trends with lifetime fertility, Supplementary Appendix Table 8 shows the lifetime mean number of children and ultimate childless levels for the Finnish cohorts 1971–1975 with full education and childbearing histories. The variation across fields is highly consistent with the variation in TFR and TFRp1 observed in 2010. Mean number of children and ultimate childlessness vary among the secondary educated from 1.48 children and one-third childless (ICT) to 2.32 children and 12% childless (health and welfare). Similarly, at higher tertiary level, the women educated in ICT and arts and humanities had 1.59 children on average and one-fourth remained childless, and the women educated in health, welfare, and education had more than two children and 12–13% remained childless. This variation in lifetime fertility is also similar to patterns observed in Sweden (Hoem, Neyer and Andersson, 2006a, 2006b).

To gain an overview of the process of changing the educational field and its consequences on fertility variation by educational field, we calculated the proportion earning a degree in another field than the highest obtained degree at the time of the first birth and by the initial field for mothers and the childless. The latter is to overcome the underlying variation in the propensities to re-educate by different initial fields irrespective of children. The overall propensity to change field after the first birth is higher among the lower educated, but regardless of educational level, the propensity to change field is the highest in arts and humanities, ICT, services, and natural sciences and the lowest in health, welfare, and education. Furthermore, those that change the field re-educate most typically in health, followed by business. However, re-educating in another field after a secondary-level degree is higher among mothers than among childless women in all fields but health. On the contrary, at the tertiary levels, childless women are more prone to change their field than mothers, and those initially educated in teaching and health are particularly more likely to attain a degree in another field if they are childless compared with mothers. These findings suggest that work in fields such as health, welfare, and education is indeed more compatible with childbearing.

## Discussion

This study examined how the fertility decline in the 2010s in Finland is related to the field of study and the economic uncertainty of the field. Calculating the change in TFRs and the expected share ever having a first birth (TFRp1) in the 2010s for 153 educational groups showed declines across (nearly) all fields, but considerable variation in the strength of the declines. Stronger declines (around −40% in TFR and −30% in TFRp1) were found not only in fields with initially lower levels, such as ICT, arts and humanities, and general education but also in natural science and engineering with more average levels. Weaker declines (around −20% in TFR and −10% in TFRp1) were observed not only in fields with initially higher levels (health and teaching) but also in agriculture. In absolute terms, the declines across fields were more similar in total fertility, but variation was notable in the case of first births. Furthermore, we observed that the fertility decline was stronger in fields characterized by higher unemployment levels, lower shares working in the public sector, and lower income levels. Together, these uncertainty measures explained one-fourth of the variation in the decline in total fertility and two-fifths in the decline in first births. The results indicate that the fertility declines have been milder among women educated in fields characterized by lower levels of economic uncertainty.

Fertility has for several decades been relatively high in the Nordic countries, but the variation in fertility across fields of education over the past decades has raised concern and is often seen as an important policy challenge in the Nordic countries, given that gender equality in the family and in the public sphere is an explicit policy goal (Rønsen and Skrede, 2010). We now observed evidence of diverging fertility levels across fields of education, especially for first births. Previous studies have shown that the recent decline in the Nordic countries may result not only in later but also in less eventual childbearing (Hellstrand *et al.*, 2021). Hence, it is plausible that the diverging period fertility trends found in this study may turn into diverging cohort patterns in fertility across study fields.

The findings of this study add to the previous evidence showing accelerated first-birth decline among the least educated (Comolli *et al.*, 2021) and among those with weaker labour market attachment (Ohlsson Wijk and Andersson, 2022). They highlight that first births are being increasingly postponed or forgone also by those with a degree but educated in fields characterized by higher economic uncertainty. Before the onset of the decline, the positive gradient in childlessness across educational levels was turning negative (Jalovaara *et al.*, 2019). The current findings provide further support for the claim that social inequality in childbearing is growing in the Nordic countries. This illustrates what might be the downside of strong work–family reconciliation policies and the dual-earner model; a strong labour market position seems to have increasingly become a prerequisite for childbearing among both men and women, implying that those with a weaker labour market position are facing growing difficulties in family formation.

Scholars highlight perceived uncertainty, which makes the future less predictable (Vignoli *et al.*, 2020a, 2020b) and more difficult to control by one’s own means (Neyer *et al.*, 2022), as a factor explaining fertility change in the 2010s. The current results indicate that objective uncertainty also mattered to some extent for the recent fertility declines: women educated in fields with less certain labour market prospects seem to have become more sensitive to their circumstances. Perceived uncertainties together with a changing labour market (e.g., increased globalization and automation) (Mills and Blossfeld, 2005; Sutela, Pärnänen and Keyriläinen, 2019), weaker income growth among the lower paid (OECD, 2020), and rising living costs (especially rents and house prices) (Eurostat, 2022a; OECD, 2023) may have contributed to particular difficulties among those with more objectively uncertain employment prospects in realizing childbearing plans.

Fertility declines also witnessed in fields with less uncertain labour market prospects indicate that there are clearly other factors besides those related to labour market uncertainties underlying the recent fertility decline. For instance, although the fields of health and teaching typically experienced weaker declines in first births, some fields (e.g., pre-school teachers and social workers) had mainly average declines in total fertility. This could reflect that in times perceived as increasingly uncertain, family-oriented women with a strong desire to become mothers rather decide to lower their number of children than to fully forego motherhood. In addition, some fields might be more affected by uncertainties other than economic ones. For instance, women educated in natural sciences experienced relatively strong declines—much stronger than those predicted by the uncertainty model—and women educated in these fields may be more strongly aware of climate change, for instance, which might make them hesitant to have children. Moreover, changes in union patterns may have contributed differently to the decline across fields, although most of the decline is attributable to declines in fertility among couples (Hellstrand, Nisén and Myrskylä, 2022).

This study has several limitations. We were unable to include partners’ characteristics since this would have implied excluding single individuals, which is not optimal in analysing aggregate fertility trends. Furthermore, this study was focused on women. We would expect stronger fertility declines in fields with higher unemployment and lower income levels also among men (Hellstrand, Nisén and Myrskylä, 2022), but the role of the public sector for the declines may be gender specific, given that men much less often work in this sector (Official Statistics of Finland (OSF), 2022). Finally, our study was unable to measure potential changes in childbearing preferences across fields, although they have been suggested to fuel the recent decline (Golovina *et al.*, 2023). Our additional analyses show that the fertility decline was weaker in fields with a higher share of females. This may indicate weaker declines when the social environment is more supportive of childbearing and less competitive, or alternatively, the increasing importance of preferences for having children during a period of increased uncertainty. Some suggest a change in the cultural landscape of childbearing in the sense that childbearing norms have weakened (Rotkirch, 2020), and one suggested mechanism behind this change is increased social media use (Tanskanen, 2018), as frequent social media use could mean time displacement from other activates and higher exposure to different lifestyles (Savelieva, Jokela and Rotkirch, 2023). Further empirical studies are needed to better understand the current behavioural change.

All measures included in the uncertainty model were hypothesized to capture aspects of economic uncertainty, and all measures were associated with the fertility decline in the bivariate analyses. However, it should be noted that shifting from the bivariate analyses to the (multivariate) uncertainty model—and further to sensitivity analysis taking additional factors into account—only unemployment was consistently and substantially associated with the heterogeneity across educational groups in the fertility decline. Much of the variation in income was captured by unemployment, and public sector work turned insignificant when factors like the share females or the share married in the field—factors that could be considered proxies for family orientation—were added to the model. Unemployment alone explained 17% and 37% of the variation in the decline in TFR and TFRp1, respectively, which is only slightly less than the full uncertainty model. For this reason, we believe that economic uncertainty is one important factor behind the decline, especially in the case of the first births, although it is certainly not the only one.

To conclude, this study provided insights into the strong and unexpected fertility decline in the 2010s in Finland. The results showed that fertility declined in (nearly) all fields of study, but that there was notable variation in the decline across fields, with more pronounced declines in first births in fields characterized by higher economic uncertainty. This implies that the social divergence in fertility is growing. Policy measures should aim at alleviating the barriers to childbearing, which in contemporary Finland is mainly not related to the incompatibility of a well-established career with childbearing, but more often to the lack of a well-established career.

## Supplementary data

Supplementary data are available at *ESR* online.

jcae001_suppl_Supplementary_Appendix

## Data Availability

The data underlying this article were provided by Statistics Finland (license number TK/780/07.03.00/2020-4). Individual-level register data from Statistics Finland are not freely available.

## Footnotes

1. This corresponds to approximately 50 fields of education per education level beyond the primary level.

2. UAS provides more practical education than universities, they do not offer postgraduate degrees, and UAS Master’s degrees require some prior work experience before acceptance.

3. This refers to total earned income subject to state taxation.

4. Employed students who received student benefits were not included when these characteristics were calculated.

5. The analyses answer the question of how current characteristics of the fields reflecting uncertainty are associated with fertility declines of a field, but sensitivity analyses were performed using characteristics from the peak fertility year (2010) and the changes in the characteristics by field over the decade.

6. The correlation between unemployment and income is −0.56 (results not shown).

7. Being enrolled in education, which we measure by proportion of students, lowers the risk of childbearing transitions, and enrolment rates differ by field of education (e.g. Lappegård and Rønsen, 2005).

8. Further stratifying the results by broad fields—health and teaching, which could be considered a selected group with higher childbearing preferences (e.g. Hoem, Neyer and Andersson, 2006a), versus all other fields—shows that the association between unemployment and the first birth decline remains significant for both groups, but the association between unemployment and the TFR decline remains significant only for fields outside health and teaching (Supplementary Appendix Table 7). Public sector work is no longer significant for any of the groups, but the estimate remains similar for the decline in first birth in health and teaching.

## Notes

**Julia Hellstrand** is a post-doctoral researcher at the University of Helsinki. Current research interests comprise fertility and family dynamics. Her work has been published in journals such as *Demography*, *European Journal of Population*, and *Population Studies*.

**Jessica Nisén** is a post-doctoral researcher and docent in sociology (especially demography) at the University of Turku. Her research interests comprise fertility and family demographics, as well as labour market impacts of fertility. Common themes in her work are gender, stratification and life course. Her work has been published in journals such as *Advances in Life Course Research*, *European Journal of Population*, and *Demographic Research*.

**Mikko Myrskylä** is Director of the Max Planck Institute for Demographic Research and Professor of Social Data Science at the University of Helsinki. Current research interests comprise population health, fertility dynamics, and demographic forecasting. His work has been published in journals such as *Nature*, *Demography*, and *Population and Development Review*.
