# Supplementary material for: Educational field, economic uncertainty, and fertility decline in Finland in 2010–2019
Source: Eur Sociol Rev. 2024 Jan 31;40(5):754–71. doi: 10.1093/esr/jcae001 (PMC11451949; doi:10.1093/esr/jcae001)
Supplement: jcae001_suppl_Supplementary_Appendix [file jcae001_suppl_supplementary_appendix.pdf]

Educational field, economic uncertainty, and fertility decline in Finland in 2010–2019

Appendix tables and figures

Appendix Table 1: TFR by field of education in 2009–2011 and 2017–2019, and the change between these two periods.

	N2017–19 (15–49)	N2018 (30–34)	TFR 2009–11	TFR 2017–19	Change, %
**Arts & humanities**					
**Higher tertiary**					
Speech science	4537	401	2.07	1.83	-11.6
Finnish language	9021	606	1.99	1.29	-35.2
Religion, theology, philosophy, & ethics	6751	493	1.92	1.46	-24
Music & performing arts	8518	530	1.81	1.36	-24.9
Interdisciplinary fields in art	9175	573	1.73	1.12	-35.3
Swedish language	6347	456	1.70	1.37	-19.4
Languages (excluding Finnish and Swedish)	18107	1243	1.70	1.19	-30
History & archeology	7641	508	1.67	1.10	-34.1
Literature & linguistics	6167	424	1.61	1.17	-27.3
Translator (excluding Swedish)	5113	351	1.52	1.04	-31.6
Cultural studies	5345	358	1.29	1.12	-13.2
Fine arts	2878	163	1.28	0.85	-33.6
**Lower tertiary**					
Translator (UAS or similar)	2095	179	1.69	1.54	-8.9
Fashion, interior and industrial design	12222	1079	1.61	1.18	-26.7
Interdisciplinary fields in art	13620	873	1.59	0.89	-44
Audio-visual techniques and media production	12143	1064	1.47	0.88	-40.1
Music & performing arts	3868	299	1.45	1.01	-30.3
Humanities (excluding history and languages)	1879	77	1.42	0.91	-35.9
Languages (excluding Finnish)	7649	416	1.27	0.89	-29.9
Fine arts	3925	306	1.21	0.72	-40.5
History & archeology	3301	175	1.04	0.69	-33.7

Finnish language	1694	76	0.99	0.93	-6.1
Literature & linguistics	1607	92	0.88	0.84	-4.5
**Secondary**					
Handicrafts	27177	1874	1.61	1.10	-31.7
Fashion, interior and industrial design	8126	385	1.59	1.27	-20.1
Music & performing arts	3475	155	1.57	0.93	-40.8
Audio-visual techniques and media production	17241	913	1.47	0.83	-43.5
Fine arts	5273	292	1.13	0.59	-47.8
**Business, law, & social sciences**					
**Higher tertiary**					
Psychology	13312	827	2.02	1.55	-23.3
Interdisciplinary fields in business	55305	3790	1.99	1.40	-29.6
Civics	5761	393	1.92	1.26	-34.4
Interdisciplinary fields in social sciences	5702	368	1.83	1.26	-31.1
Sociology	19999	1279	1.81	1.39	-23.2
Law	17213	1327	1.73	1.48	-14.5
Economics	1825	105	1.69	1.31	-22.5
Politics	8285	577	1.68	1.36	-19
Journalism & reporting	7668	460	1.61	1.22	-24.2
**Lower tertiary**					
Interdisciplinary fields in business: UAS	182172	8302	1.71	1.26	-26.3
Secretarial and office work	6591	513	1.60	1.12	-30
Journalism & reporting	2190	109	1.39	0.64	-54
Interdisciplinary fields in business: University	6814	188	1.28	0.92	-28.1
Library, information, & archival studies	2250	141	1.15	0.52	-54.8
Sociology & psychology	6250	267	1.15	0.81	-29.6
Law	2459	63	1.14	0.74	-35.1
Politics & civics	3181	164	1.13	0.90	-20.4
**Secondary**					
Interdisciplinary fields in business	130139	7224	1.79	1.31	-26.8
Marketing and advertisement	3068	198	1.73	1.14	-34.1
Wholesale and retail sales	25517	1605	1.68	1.34	-20.2

Secretarial and office work	5919	385	1.56	1.19	-23.7
Accounting and taxation	3774	200	1.53	0.97	-36.6
Management and administration	6454	332	1.18	0.90	-23.7
**Engineering, agriculture, ICT, & natural sciences**					
**Higher tertiary**					
Electricity, energy, electronics, & automation	4382	284	2.07	1.27	-38.6
Building and civil engineering	3705	245	2.05	1.41	-31.2
Chemical engineering and processes	4582	294	2.04	1.47	-27.9
Veterinary	3248	230	2.04	1.44	-29.4
Mathematics and statistics	5893	458	2.03	1.79	-11.8
Interdisciplinary fields in processing and manufacturing	3658	245	1.94	1.38	-28.9
Interdisciplinary fields in engineering	10632	740	1.93	1.39	-28
Interdisciplinary fields in agriculture	3483	264	1.92	1.41	-26.6
Biochemistry	4370	321	1.89	1.23	-34.9
Earth science	5802	391	1.87	1.17	-37.4
Crop and livestock production	2271	148	1.85	1.64	-11.4
Chemistry	5060	262	1.83	1.12	-38.8
Forestry	2055	129	1.80	1.46	-18.9
Environmental studies	3678	205	1.80	1.15	-36.1
Biology	8874	590	1.76	1.15	-34.7
Architecture and town planning	4126	339	1.70	1.37	-19.4
Information and communication technologies	8145	362	1.67	1.10	-34.1
Physics	1972	124	1.51	0.99	-34.4
**Lower tertiary**					
Crop and livestock production	8001	517	2.17	1.77	-18.4
Interdisciplinary fields in engineering	7578	598	1.93	1.27	-34.2
Building and civil engineering	7714	580	1.86	1.44	-22.6
Electricity, energy, electronics, & automation	3111	138	1.84	1.13	-38.6
Chemical engineering and processes	9017	608	1.83	1.19	-35
Interdisciplinary fields in processing and manufacturing	5046	241	1.78	1.04	-41.6

Mechanics and metal trades	2464	181	1.78	1.16	-34.8
Forestry	3348	212	1.68	1.17	-30.4
Environmental protection technology	4117	308	1.68	1.19	-29.2
Information and communication technologies	20039	763	1.62	0.86	-46.9
Horticulture	2907	191	1.57	1.43	-8.9
Architecture and town planning	2054	79	1.56	1.05	-32.7
Interdisciplinary fields in natural sciences	6612	323	1.44	0.91	-36.8
**Secondary**					
Food processing	24711	1310	2.26	1.56	-31
Chemical engineering and processes	8806	499	1.83	1.06	-42.1
Horticulture	14314	741	1.80	1.36	-24.4
Materials (glass, paper, plastic, and wood)	15920	1048	1.80	1.14	-36.7
Crop and livestock production	29639	1702	1.75	1.35	-22.9
Building and civil engineering	9815	526	1.72	1.34	-22.1
Textiles (clothes, footwear, and leather)	19558	954	1.68	1.23	-26.8
Mechanics and metal trades	4417	244	1.62	1.21	-25.3
Natural environments and wildlife	3142	239	1.59	1.02	-35.8
Motor vehicles, ships, and aircraft	5818	304	1.49	1.31	-12.1
Electricity, energy, electronics, & automation	4204	240	1.48	1.19	-19.6
Information and communication technologies	7535	416	1.42	0.88	-38
**Health, welfare, & teaching**					
**Higher tertiary**					
Teacher (without subject specification)	38617	2253	2.63	1.94	-26.2
Teacher: home economics	2481	166	2.51	1.93	-23.1
Teacher: study adviser, music, other	1910	142	2.49	1.59	-36.1
Medicine	23442	1767	2.45	1.93	-21.2
Education science: early childhood education	3170	228	2.44	1.90	-22.1
Special teacher	3564	217	2.28	1.85	-18.9
Pharmacy	4454	295	2.10	1.57	-25.2
Dental studies	4329	389	2.09	1.89	-9.6
Education science (excluding early childhood education)	16408	874	2.08	1.53	-26.4

Teacher: handicrafts	2439	160	1.91	1.57	-17.8
Interdisciplinary studies in health & welfare	24984	1164	1.73	1.58	-8.7
**Lower tertiary**					
Midwife	8822	652	2.61	2.08	-20.3
Health care provider	33559	2539	2.43	1.88	-22.6
Optics	2901	173	2.29	1.63	-28.8
Dental studies	5519	389	2.28	1.66	-27.2
Pre-school teacher	16190	749	2.22	1.64	-26.1
Nurse	114318	7007	2.18	1.71	-21.6
Physical therapy	25565	1903	2.16	1.62	-25
Social work	78941	5541	2.10	1.54	-26.7
Pharmacy	11361	600	2.05	1.61	-21.5
Occupational therapy	2434	113	1.91	1.06	-44.5
Laboratory	8515	619	1.89	1.44	-23.8
X-ray and other technical matters	5669	458	1.86	1.28	-31.2
Interdisciplinary studies in health & welfare	6925	279	1.84	1.27	-31
Teacher (with subject specification)	6389	428	1.77	1.27	-28.2
Education science	7769	257	1.59	1.16	-27
**Secondary**					
Nurse	237972	13897	2.28	1.77	-22.4
Child care	25455	1278	2.10	1.68	-20
Pharmacy	3165	186	1.81	1.53	-15.5
Youth services	10377	609	1.81	1.35	-25.4
Therapy & rehabilitation (massage and pedicure)	11136	545	1.70	1.21	-28.8
School care	17559	1014	1.67	1.20	-28.1
Interdisciplinary studies in health & welfare	5556	183	1.66	1.53	-7.8
Medical diagnostic & treatment (equipment maintenance)	2717	140	1.07	0.84	-21.5
**Services**					
**Higher tertiary**					
Sports	3407	241	2.34	1.43	-38.9

**Lower tertiary**					
Hotel & business	11136	810	2.09	1.21	-42.1
Sports	3809	241	1.93	1.21	-37.3
Hair & beauty services	2614	185	1.88	1.14	-39.4
Hotel & catering	19002	885	1.73	1.17	-32.4
Tourism	21810	1803	1.65	1.16	-29.7
Domestic services	3987	32	1.63	1.15	-29.4
Interdisciplinary fields in services	1814	113	1.44	0.94	-34.7
**Secondary**					
Restaurant & catering	35044	2872	1.91	1.63	-14.7
Hairdresser	37675	2301	1.87	1.40	-25.1
Hotel, restaurant, & catering	78212	4291	1.82	1.36	-25.3
Beautician	16708	921	1.78	1.28	-28.1
Transport services	8242	420	1.72	1.22	-29.1
Security services	8959	466	1.69	1.38	-18.3
Tourism	15040	881	1.67	1.17	-29.9
Domestic services	37034	1336	1.64	1.12	-31.7
Waitress	4203	108	1.58	1.11	-29.7
Sales and customer services in hotels	3127	55	1.45	0.62	-57.2
Sports	5284	233	1.36	1.25	-8.1
Chef	14304	159	1.36	1.00	-26.5
**General/other**					
**Higher tertiary**					
Other	4557	270	1.22	1.00	-18
**Lower tertiary**					
Other	3459	174	1.14	0.88	-22.8
**Secondary**					
Other	6962	287	1.76	1.34	-23.9
General	327556	7380	1.35	0.91	-32.6
**Primary**					
General	502306	9148	1.72	1.24	-27.9

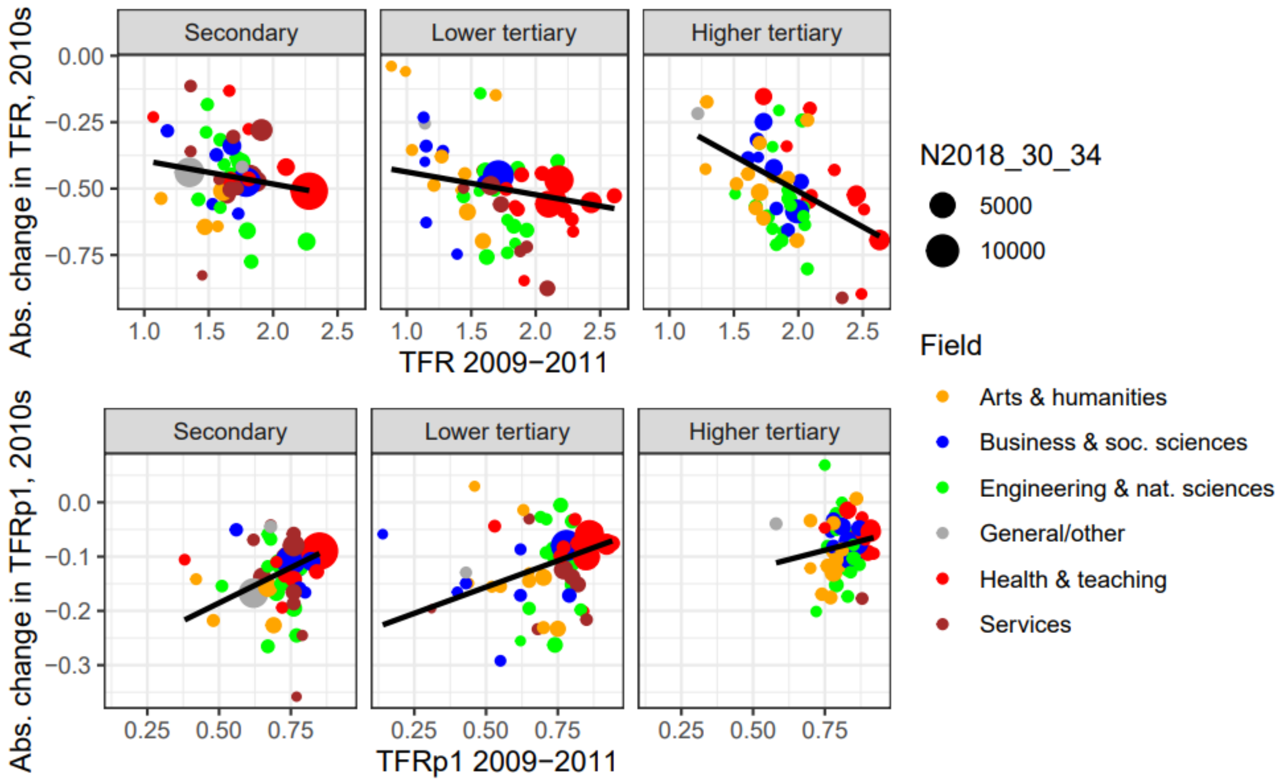

Appendix Figure 1: Top panels: TFR in 2009–2011 and absolute change in TFR in the 2010s by level and field of education. Bottom panels: TFRp1 in 2009–2011 and absolute change in TFRp1 in the 2010s by level and field of education.

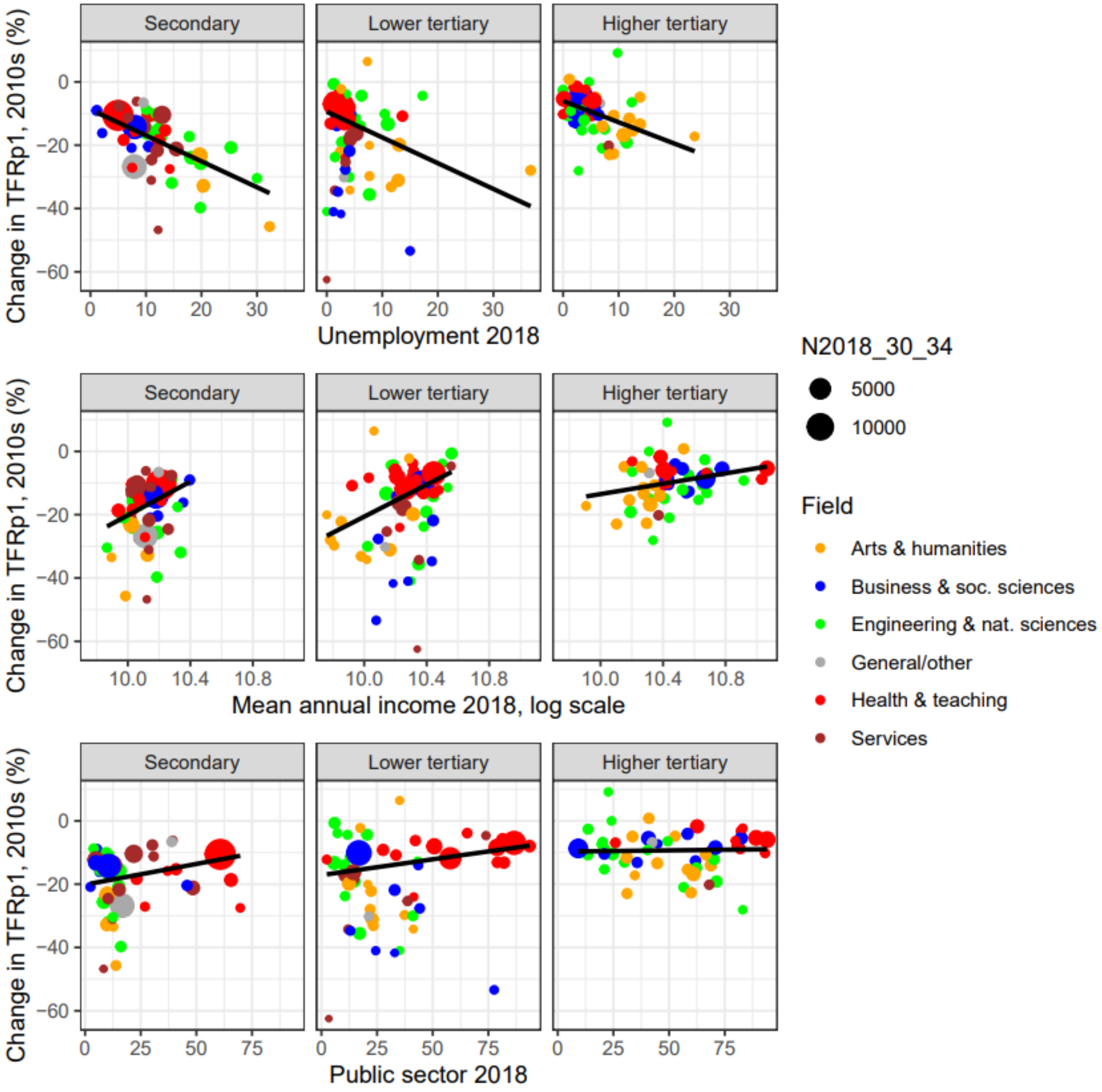

Appendix Figure 2: Uncertainty measures and the relative decline in TFRp1 in the 2010s.

Appendix Table 2: Regression models estimating the relative change in TFR and TFRp1 in the 2010s. Sensitivity checks: Characteristics measured in 2010 and the changes in characteristics in 2010-2018. The models are weighted by size of the field. Number of fields: 152.

	**Change in TFR (%)**			**Change in TFRp1 (%)**		
	Original results	Sensitivity: Characteristics measured in 2010	Sensitivity: The change in characteristics in 2010–2018	Original results	Sensitivity: Characteristics measured in 2010	Sensitivity: The change in characteristics in 2010–2018
**Intercept**	0.36***	0.40***	0.05	0.17*	0.22*	-0.11
**Unemployment**	-0.33***	-0.23**	-0.33**	-0.36***	-0.21**	-0.31***
**log(Income)**	0.00	0.05	-0.16	0.07	0.15	-0.06
**Public sector**	0.19***	0.18**	0.21***	0.12**	0.13**	0.16***
**Unemployment change**			-0.02			-0.05*
**Income change**			0.00			0.00
**Public change**			0.00			-0.01
**Lower tertiary**	-0.54***	-0.52***	-0.46**	-0.11	-0.07	0.02
**Higher tertiary**	-0.35	-0.40	-0.16	0.13	0.04	0.31
R^2	0.24	0.19	0.26	0.40	0.32	0.43
**Adjusted** R^2	0.22	0.17	0.22	0.38	0.30	0.40

The significance levels at 0.001, 0.01 and 0.05 are depicted by ***, **, and * respectively.

Appendix Table 3: Regression models estimating the relative change in TFR and TFRp1 in the 2010s. Sensitivity checks: the characteristics for secondary educated are measured at age 20-24 whenever possible. The models are weighted by size of the field. Number of fields: 152.

	**Change in TFR (%)**		**Change in TFRp1 (%)**	
	Original results	Sensitivity: Characteristics at age 20-24 for secondary educated	Original results	Sensitivity: Characteristics at age 20-24 for secondary educated
**Intercept**	0.36***	0.54***	0.17*	0.39***
**Unemployment**	-0.33***	-0.26**	-0.36***	-0.26***
**log(Income)**	0.00	0.17	0.07	0.28***
**Public sector**	0.19***	0.18***	0.12**	0.11*
**Lower tertiary**	-0.54***	-0.73***	-0.11	-0.33**
**Higher tertiary**	-0.35	-0.68***	0.13	-0.26
R^2	0.24	0.26	0.40	0.43
**Adjusted** R^2	0.22	0.23	0.38	0.41

The significance levels at 0.001, 0.01 and 0.05 are depicted by ***, **, and * respectively.

Appendix Table 4: Uncertainty model + additional factors explaining the change in TFR. Number of fields: 152.

	**Change in TFR**								
	Separate models		Uncertainty model, M	M + Student	M + Occupational match	M + Share females	M + Single	M + Cohabitation	M + Married
	**Est.**	$\mathbf{R^2}$	**Est.**	**Est.**	**Est.**	**Est.**	**Est.**	**Est.**	**Est.**
**Intercept**			0.36***	0.26**	0.35***	0.36***	0.24**	0.34***	0.24**
**Unemployment**	-0.40***	0.17	-0.33***	-0.36***	-0.30***	-0.28**	-0.20*	-0.27**	-0.27**
**log(Income)**	0.23**	0.06	0.00	-0.12	-0.08	0.11	-0.09	-0.04	-0.06
**Public sector**	0.25***	0.14	0.19***	0.15**	0.13*	0.10	0.07	0.20***	-0.01
**Student**	-0.40***	0.13		-0.33***					
**Occupational match**	0.27***	0.17			0.17**				
**Share females**	0.29***	0.12				0.22**			
**Single**	-0.43***	0.30					-0.33***		
**Cohabitation**	0.24***	0.09						0.16*	
**Married**	0.40***	0.27							0.34***
**Lower tertiary**			-0.54***	-0.45***	-0.58***	-0.58***	-0.49***	-0.56***	-0.43**
**Higher tertiary**			-0.35	-0.32	-0.32	-0.42*	-0.16	-0.28	-0.20
R^2			0.24	0.31	0.29	0.28	0.33	0.27	0.33
**Adjusted** R^2			0.22	0.28	0.26	0.25	0.31	0.24	0.30

Similarly as for the uncertainty measures, the additional factors are measured at age 25–29. The significance levels at 0.001, 0.01 and 0.05 are depicted by ***, **, and * respectively.

Appendix Table 5: Uncertainty model + additional factors explaining the change in TFRp1. Number of fields: 152.

	**Change in TFRp1**								
	Separate models		Uncertainty model, M	M + Student	M + Occupational match	M + Share females	M + Single	M + Cohabitation	M + Married
	**Est.**	$\mathbf{R^2}$	**Est.**	**Est.**	**Est.**	**Est.**	**Est.**	**Est.**	**Est.**
**Intercept**			0.17*	0.01	0.15*	0.17*	-0.01	0.11	0.03
**Unemployment**	-0.45***	0.37	-0.36***	-0.42***	-0.32***	-0.31***	-0.18**	-0.23**	-0.30***
**log(Income)**	0.32***	0.23	0.07	-0.11	-0.06	0.18*	-0.07	-0.01	0.00
**Public sector**	0.19***	0.23	0.12**	0.06	0.03	0.03	-0.06	0.15***	-0.10
**Student**	-0.57***	0.42		-0.52***					
**Occupational match**	0.36***	0.45			0.28***				
**Share females**	0.24***	0.23				0.23***			
**Single**	-0.51***	0.61					-0.49***		
**Cohabitation**	0.41***	0.39						0.33***	
**Married**	0.40***	0.43							0.39***
**Lower tertiary**			-0.11	0.03	-0.18	-0.15	-0.03	-0.15	0.02
**Higher tertiary**			0.13	0.17	0.17	0.05	0.41**	0.27	0.30*
R^2			0.40	0.60	0.54	0.45	0.63	0.52	0.53
**Adjusted** R^2			0.38	0.58	0.52	0.42	0.62	0.50	0.51

Similarly as for the uncertainty measures, the additional factors are measured at age 25–29. The significance levels at 0.001, 0.01 and 0.05 are depicted by ***, **, and * respectively.

Appendix Table 6: Regression models estimating the relative change in TFR and TFRp1 in the 2010s. Sensitivity checks: Analyses stratified by female composition (the standardized share females >=0 versus <0). The models are weighted by size of the field. Number of fields: 152.

	**Change in TFR (%)**			**Change in TFRp1 (%)**		
	Original results	Females >=0	Females <0	Original	Females >=0	Females <0
**Intercept**	0.36***	0.58***	0.25	0.17*	0.28**	-0.14
**Unemployment**	-0.33***	-0.22	-0.50***	-0.36***	-0.36***	-0.36*
**log(Income)**	0.00	0.20	-0.01	0.07	0.02	0.24
**Public sector**	0.19***	0.10	0.19	0.12**	0.11*	-0.10
**Lower tertiary**	-0.54***	-0.63***	-0.53*	-0.11	-0.21	-0.02
**Higher tertiary**	-0.35	-0.47	-0.50	0.13	0.10	0.10
R^2	0.24	0.23	0.25	0.40	0.45	0.41
**Adjusted** R^2	0.22	0.18	0.18	0.38	0.42	0.36

The significance levels at 0.001, 0.01 and 0.05 are depicted by ***, **, and * respectively.

Appendix Table 7: Regression models estimating the relative change in TFR and TFRp1 in the 2010s. Sensitivity checks: Analyses stratified by fields in health and teaching, and other fields. The models are weighted by size of the field. Number of fields: 152.

	**Change in TFR (%)**			**Change in TFRp1 (%)**		
	Original results	Health & teaching	Other fields	Original	Health & teaching	Other fields
**Intercept**	0.36***	0.51*	0.26	0.17*	0.17	-0.07
**Unemployment**	-0.33***	-0.25	-0.33**	-0.36***	-0.34*	-0.30***
**log(Income)**	0.00	0.07	-0.06	0.07	-0.07	0.10
**Public sector**	0.19***	0.05	0.10	0.12**	0.12	-0.11
**Lower tertiary**	-0.54***	-0.38	-0.56**	-0.11	0.05	-0.11
**Higher tertiary**	-0.35	-0.15	-0.22	0.13	0.37*	0.41
R^2	0.24	0.19	0.14	0.40	0.51	0.32
**Adjusted** R^2	0.22	0.05	0.10	0.38	0.43	0.29

The significance levels at 0.001, 0.01 and 0.05 are depicted by ***, **, and * respectively.

Appendix Table 8: Mean number of children, % childless, % changing field of education after first birth by age 44 years, % changing field of education after the first initial field of education at respective level, Finnish native women born in 1971–1975.

	N (44)	Mean number of children	% childless	N (first birth)	Change field after FB	Distribution of new fields (top 3)	N (first initial field)	Change field, parents	Change field, childless
**Primary**	8379	2.07	23.2	14493	55.7	health 49%, business 20%, services 18%	19113	58.0	45.5
**Secondary**									
**Health & welfare**	15383	2.32	11.9	8210	9.9	business 37%, services 17%, education 11%	9341	10.0	16.3
**Services**	12462	1.97	21.0	12144	36.8	health 60%, business 24%, engineering 6%	21064	51.8	45.0
**Engineering**	4280	1.86	23.7	4149	42.2	health 49%, business 24%, services 16%	5798	51.8	47.2
**Business**	7667	1.86	19.5	3561	25.3	health 60%, services 18%, engineering 6%	6414	30.1	24.9
**Agriculture**	1831	1.84	25.3	1385	36.2	health 43%, business 24%, services 11%	1704	45.0	42.5
**Arts & humanities**	2000	1.72	29.2	1654	41.1	health 43%, business 26%, services 11%	2176	54.2	43.6
**General**	4302	1.57	31.2	8855	69.2	health 29%, business 19%, arts & hum. 16%	72143	94.8	90.9
**ICT**	383	1.48	33.7	170	41.8	health 38%, business 31%, engineering 13%	226	51.2	32.8
**Lower tertiary**									
**Education**	1777	2.18	12.0	1619	5.3	arts & hum. 57%, health 16%, soc. sciences 15%	2696	8.0	14.2
**Health & welfare**	18677	2.12	13.1	13306	5.3	soc. sciences 38%, education 26%, arts & hum. 12%	20968	7.6	12.0
**Agriculture**	975	1.89	22.5	688	9.4	health 25%, business 22%, engineering 15%	1067	12.7	14.6
**Services**	4006	1.79	19.9	3161	12.3	health 37%, business 26%, education 18%	4803	20.4	21.4
**Engineering**	2225	1.71	22.0	1585	7.9	health 30%, business 26%, nat. sciences 11%	2568	14.1	14.3
**Business**	16886	1.69	22.2	13067	7.4	health 36%, soc. sciences 14%, arts & hum. 13%	21164	16.5	17.1
**ICT**	1657	1.63	23.1	1152	10.8	business 46%, health 23%, education 11%	1510	21.3	21.1
**Social sciences**	506	1.47	30.2	266	8.3	business 36%, education 23%, arts & hum. 18%	789	14.9	15.6
**Arts & humanities**	2513	1.42	32.0	1613	11.8	education 29%, health 27%, business 15%	4297	12.5	9.7
**Natural sciences**	101	1.24	40.6	42	19.0	health 50%	278	19.0	14.5
**Higher tertiary**									
**Education**	5172	2.13	12.4	3153	1.9	arts & hum. 36%, soc. sciences 34%, health 12%	5198	2.7	5.6
**Health & welfare**	4188	2.03	13.2	1752	1.2	business 29%, soc. sciences 19%, arts & hum. 14%	3967	1.5	3.8

**Engineering**	1982	1.87	17.0	1275	2.9	business 49%, ICT 19%, soc. sciences 14%	1976	4.7	6.1
**Agriculture**	799	1.83	18.4	498	3.6	business 28%, education 22%, health 22%	792	5.3	6.2
**Natural sciences**	2730	1.77	21.1	1853	8.8	health 62%, engineering 10%, agriculture 10%	3060	12.7	10.8
**Business**	4542	1.72	18.8	2611	1.3	soc. sciences 26%, arts & hum. 21%, education 21%	4398	1.9	2.9
**Services**	410	1.68	22.7	167	1.8	-	400	2.6	0.0
**Social sciences**	4475	1.67	20.3	2469	2.8	business 36%, education 24%, arts & hum. 17%	4409	4.1	5.0
**Arts & humanities**	6466	1.59	25.4	3451	2.6	soc. sciences 42%, education 23%, business 14%	6606	3.9	4.8
**ICT**	517	1.59	25.2	228	5.3	engineering 33%, soc. sciences 25%)	461	4.0	8.0
